# Supplementary material for: Genetic diversity and connectivity of the megamouth shark (Megachasma pelagios)
Source: PeerJ. 2018 Mar 5;6:e4432. doi: 10.7717/peerj.4432 (PMC5842762; doi:10.7717/peerj.4432)
Supplement: Table S1 [file peerj-06-4432-s003.docx]

Table S1. Megamouth sharks recorded to date.

| Number | Capture location | Capture date | Reference |
| --- | --- | --- | --- |
| No. 1 | Hawaii, USA | 15-Nov-76 | Nakaya 2010 |
| No. 2 | California, USA | 29-Nov-84 | Nakaya 2010 |
| No. 3 | Mandurah, W. Australia | 18-Aug-88 | Nakaya 2010 |
| No. 4 | Shizuoka, Japan | 23-Jan-89 | Nakaya 2010 |
| No. 5 | Shizuoka, Japan | 12-Jun-89 | Nakaya 2010 |
| No. 6 | California, USA | 21-Oct-90 | Nakaya 2010 |
| No. 7 | Fukuoka, Japan | 29-Nov-94 | Nakaya 2010 |
| No. 8 | Dakar, Senegal | 4-May-95 | Nakaya 2010 |
| No. 9 | Southern Brazil | 18-Sep-95 | Nakaya 2010 |
| No. 10 | Mie, Japan | 30-Apr-97 | Nakaya 2010 |
| No. 11 | Cagayan de Oro, | 21-Feb-98 | Nakaya 2010 |
| No. 12 | Mie, Japan | 23-Apr-98 | Nakaya 2010 |
| No. 13 | Manado, Indonesia | 30-Aug-98 | Nakaya 2010 |
| No. 14 | California, USA | 1-Oct-99 | Nakaya 2010 |
| No. 15 | California, USA | 19-Oct-01 | Nakaya 2010 |
| No. 16 | East Indian Ocean | 18-Jan-02 | Nakaya 2010 |
| No. 17 | Western Cape, South Africa | 20-Apr-02 | Nakaya 2010 |
| No. 18 | Cagayan de Oro, | 6-Jan-03 | Nakaya 2010 |
| No. 19 | California, USA | 26-May-03 | Nakaya 2010 |
| No. 20 | Hualien, Taiwan | 3-Jul-03 | Nakaya 2010 |
| No. 21 | Shizuoka, Japan | 7-Aug-03 | Nakaya 2010 |
| No. 22 | Guayas, Ecuador | 8-Mar-04 | Nakaya 2010 |
| No. 23 | Sumatra, Indonesia | 13-Mar-04 | Nakaya 2010 |
| No. 24 | Chiba, Japan | 19-Apr-04 | Nakaya 2010 |
| No. 25 | Shizuoka, Japan | 23-Apr-04 | Nakaya 2010 |
| No. 26 | Iloilo, Philippines | 4-Nov-04 | Nakaya 2010 |
| No. 27 | Mie, Japan | 23-Jan-05 | Nakaya 2010 |
| No. 28 | Macajalar Bay, Philippines | 30-Jan-05 | Nakaya 2010 |
| No. 29 | Hualien, Taiwan | 25-Apr-05 | Nakaya 2010 |
| No. 30 | Hualien, Taiwan | 2-May-05 | Nakaya 2010 |
| No. 31 | Hualien, Taiwan | 4-May-05 | Nakaya 2010 |
| No. 32 | Hualien, Taiwan | 5-May-05 | Nakaya 2010 |
| No. 33 | Hualien, Taiwan | 5-May-05 | Nakaya 2010 |
| No. 34 | Bayawan, Philippines | 26-Jan-06 | Nakaya 2010 |
| No. 35 | Cagayan de Oro, Philippines | 12-Mar-06 | Nakaya 2010 |
| No. 36 | Zhejiang, China | 23-Mar-06 | Nakaya 2010 |
| No. 37 | Kanagawa, Japan | 2-May-06 | Nakaya 2010 |
| No. 38 | Tortugas Bay, Mexico | 16-Nov-06 | Nakaya 2010 |
| No. 39 | Shizuoka, Japan | 7-Jun-07 | Nakaya 2010 |
| No. 40 | Ibaragi, Japan | 9-Jul-07 | Nakaya 2010 |
| No. 41 | Baja California, Mexico | 23-Sep-07 | Florida Museum |
| No. 42 | Southern Leyte, Philippines | 27-Sep-07 | Florida Museum |
| No. 43 | Taiwan | 30-Jun-08 | Florida Museum |
| No. 44 | Eastern Taiwan | 10-Jul-08 | Florida Museum |
| No. 45 | Southern Leyte, Philippines | 5-Sep-08 | Florida Museum |
| No. 46 | Philippines | 31-Mar-09 | Florida Museum |
| No. 47 | Taiwan | 9-Jun-09 | Florida Museum |
| No. 48 | Rio de Janeiro, Brazil | 9-Jul-09 | Florida Museum |
| No. 49 | Benitos Islands, Mexico | 6-Nov-09 | Florida Museum |
| No. 50 | Southeastern China | 25-Apr-10 | Florida Museum |
| No. 51 | Eastern Taiwan | 19-Jun-10 | Florida Museum |
| No. 52 | Bohol, Philippines | 10 | Florida Museum |
| No. 53 | Shizuoka prefecture, Japan | 24-Jun-11 | Florida Museum |
| No. 54 | Sagami Bay, Japan | 1-Jul-11 | Florida Museum |
| No. 55 | Sea of China | Jan-12 | Florida Museum |
| No. 56 | Negombo Harbour, Sri Lanka | 5-Jul-12 | Florida Museum |
| No. 57 | Eastern Taiwan | 16-Oct-12 | Florida Museum |
| No. 58 | Manazuru, Japan | 6-Sep-13 | Florida Museum |
| No. 59 | Hualien, Taiwan | 18-Apr-13 | Hsu et al. 2015 |
| No. 60 | Hualien, Taiwan | 28-Apr-13 | Hsu et al. 2015 |
| No. 61 | Hualien, Taiwan | 30-Apr-13 | Hsu et al. 2015 |
| No. 62 | Hualien, Taiwan | 5-May-13 | Hsu et al. 2015 |
| No. 63 | Hualien, Taiwan | 6-May-13 | Hsu et al. 2015 |
| No. 64 | Hualien, Taiwan | 6-May-13 | Hsu et al. 2015 |
| No. 65 | Hualien, Taiwan | 7-May-13 | Hsu et al. 2015 |
| No. 66 | Hualien, Taiwan | 8-May-13 | Hsu et al. 2015 |
| No. 67 | Hualien, Taiwan | 16-May-13 | Hsu et al. 2015 |
| No. 68 | Hualien, Taiwan | 18-May-13 | Hsu et al. 2015 |
| No. 69 | Hualien, Taiwan | 18-May-13 | Hsu et al. 2015 |
| No. 70 | Hualien, Taiwan | 19-May-13 | Hsu et al. 2015 |
| No. 71 | Hualien, Taiwan | 19-May-13 | Hsu et al. 2015 |
| No. 72 | Hualien, Taiwan | 21-May-13 | Hsu et al. 2015 |
| No. 73 | Hualien, Taiwan | 23-May-13 | Hsu et al. 2015 |
| No. 74 | Hualien, Taiwan | 30-May-13 | Hsu et al. 2015 |
| No. 75 | Hualien, Taiwan | 10-Jun-13 | Hsu et al. 2015 |
| No. 76 | Hualien, Taiwan | 13-Jun-13 | Hsu et al. 2015 |
| No. 77 | Hualien, Taiwan | 10-Jul-13 | Hsu et al. 2015 |
| No. 78 | Hualien, Taiwan | 10-Jul-13 | Hsu et al. 2015 |
| No. 79 | Hualien, Taiwan | 10-Jul-13 | Hsu et al. 2015 |
| No. 80 | Hualien, Taiwan | 17-Jul-13 | Hsu et al. 2015 |
| No. 81 | Antique, Philippines | 14-Mar-14 | Florida Museum |
| No. 82 | Shizuoka, Japan | 14-Apr-14 | Florida Museum |
| No. 83 | Hualien, Taiwan | 5-May-14 | Hsu et al. 2015 |
| No. 84 | Hualien, Taiwan | 22-May-14 | Hsu et al. 2015 |
| No. 85 | Hualien, Taiwan | 30-May-14 | Hsu et al. 2015 |
| No. 86 | Hualien, Taiwan | 31-May-14 | Hsu et al. 2015 |
| No. 87 | Hualien, Taiwan | 31-May-14 | Hsu et al. 2015 |
| No. 88 | Hualien, Taiwan | 1-Jun-14 | Hsu et al. 2015 |
| No. 89 | Hualien, Taiwan | 1-Jun-14 | Hsu et al. 2015 |
| No. 90 | Hualien, Taiwan | 4-Jun-14 | Hsu et al. 2015 |
| No. 91 | Hualien, Taiwan | 8-Jun-14 | Hsu et al. 2015 |
| No. 92 | Agusan Del Norte, Philippines | 23-Jun-14 | Florida Museum |
| No. 93 | Cagayan de Oro City, Philippines | 30-Jun-14 | Florida Museum |
| No. 94 | Hualien, Taiwan | 3-Aug-14 | Hsu et al. 2015 |
| No. 95 | Marigondon, Philippines | 28-Jan-15 | Florida Museum |
| No. 96 | Hualien, Taiwan | 14-May-15 | Hsu et al. 2015 |
| No. 97 | Hualien, Taiwan | 15-May-15 | Hsu et al. 2015 |
| No. 98 | Mie, Japan | 15-Apr-16 | Florida Museum |
| No. 99 | Puerto Rico | 10-Dec-16 | Rodriguez-Ferrer et al. 2017 |
